# Seed Priming With Protein Hydrolysates Improves Arabidopsis Growth and Stress Tolerance to Abiotic Stresses

**DOI:** 10.3389/fpls.2021.626301

**Published:** 2021-06-08

**Authors:** Mirella Sorrentino, Nuria De Diego, Lydia Ugena, Lukáš Spíchal, Luigi Lucini, Begoña Miras-Moreno, Leilei Zhang, Youssef Rouphael, Giuseppe Colla, Klára Panzarová

**Affiliations:** ^1^PSI (Photon Systems Instruments), spol. s r.o., Drásov, Czechia; ^2^Department of Agricultural Sciences, University of Naples Federico II, Naples, Italy; ^3^Centre of Region Haná for Biotechnological and Agricultural Research, Czech Advanced Technology and Research Institute, Olomouc, Czechia; ^4^Department of Chemical Biology and Genetics, Centre of the Region Haná for Biotechnological and Agricultural Research, Faculty of Science, Palacký University Olomouc, Olomouc, Czechia; ^5^Department for Sustainable Food Process - DiSTAS, Università Cattolica del Sacro Cuore, Piacenza, Italy; ^6^Department of Agriculture and Forest Sciences, University of Tuscia, Viterbo, Italy

**Keywords:** protein hydrolysates, high-throughput phenotyping, secondary metabolism, seed priming, plant biostimulant characterization index, salinity, multi- well plates

## Abstract

The use of plant biostimulants contributes to more sustainable and environmentally friendly farming techniques and offers a sustainable alternative to mitigate the adverse effects of stress. Protein hydrolysate-based biostimulants have been described to promote plant growth and reduce the negative effect of abiotic stresses in different crops. However, limited information is available about their mechanism of action, how plants perceive their application, and which metabolic pathways are activating. Here we used a multi-trait high-throughput screening approach based on simple RGB imaging and combined with untargeted metabolomics to screen and unravel the mode of action/mechanism of protein hydrolysates in Arabidopsis plants grown in optimal and in salt-stress conditions (0, 75, or 150 mM NaCl). Eleven protein hydrolysates from different protein sources were used as priming agents in Arabidopsis seeds in three different concentrations (0.001, 0.01, or 0.1 μl ml^–1^). Growth and development-related traits as early seedling establishment, growth response under stress and photosynthetic performance of the plants were dynamically scored throughout and at the end of the growth period. To effectively classify the functional properties of the 11 products a Plant Biostimulant Characterization (PBC) index was used, which helped to characterize the activity of a protein hydrolysate based on its ability to promote plant growth and mitigate stress, and to categorize the products as plant growth promoters, growth inhibitors and/or stress alleviator. Out of 11 products, two were identified as highly effective growth regulators and stress alleviators because they showed a PBC index always above 0.51. Using the untargeted metabolomics approach, we showed that plants primed with these best performing biostimulants had reduced contents of stress-related molecules (such as flavonoids and terpenoids, and some degradation/conjugation compounds of phytohormones such as cytokinins, auxins, gibberellins, etc.), which alleviated the salt stress response-related growth inhibition.

## Introduction

Nowadays, the actual yield from the main crops worldwide accounts for less than half of its potential because of the effects of abiotic stresses on plants (Bulgari et al., 2019). Among them, one of the most concerning condition is represented by salinity stress that decreases the quantity and quality of the final yield (Yamaguchi and Blumwald, 2005; Shahbaz and Ashraf, 2013), because most of the high-value agricultural crops are sensitive to salinity (Shannon and Grieve, 1999). Salinity stress generally occurs in those areas where the concentration of salt – most commonly sodium chloride (NaCl) – in the soil or in the groundwater is higher than the crop threshold sensitivity (Colla et al., 2010). This occurs especially in parts of the world where most of the agricultural areas are close to the sea, like in the Mediterranean basin (Viégas et al., 2001). Soil or water salinity can affect plants in different ways, from increasing the soil osmotic pressure to hindering the regular plant nutrition (Machado and Serralheiro, 2017). Plant biostimulants represent an eco-friendly and useful tool improving plant tolerance to abiotic adversities, like salinity (Colla et al., 2017). According to the European Biostimulant Industry Council, in the EU alone, the economic value of biostimulants is estimated to be between 200 and 400 million euros. However, despite the high economic potential of these substances, few well-characterized products are commercially available. The main problem is represented by the limited knowledge about their mode of action, mainly because they are formulated from complex, diverse, and heterogeneous materials (Brown and Saa, 2015). For this reason, plant biostimulants are usually classified more according to the plant response they cause than by their composition. In fact, “plant biostimulants” is a hypernym used to describe very different substances such as seaweed extracts, humic and fulvic acids, animal and vegetal-based protein hydrolysates, rather than microorganisms like mycorrhizal fungi and rhizospheric bacteria (Colla and Rouphael, 2015; Carillo et al., 2020). Among all the existing plant biostimulants, protein hydrolysates (PHs) are recently gaining big popularity. They are mixtures of amino acids with oligo- to polypeptides derived from the partial hydrolysis of protein-rich sources either from plant or animal origin. The application of PHs goes from foliar spray or substrate drench to adult plants (Lucini et al., 2015; Sestili et al., 2018) to seed priming, which increases abiotic stress tolerance by reprogramming the plant metabolism during the germination stages (Mahdavi, 2013; Sharma et al., 2014; Pichyangkura and Chadchawan, 2015; Van Oosten et al., 2017). Many studies have proven the efficacy of PHs in improving the quantity and quality of the yield, especially under abiotic stress or limiting conditions (Ertani et al., 2009; Colla et al., 2015; du Jardin, 2015). Indeed, they have been reported to exert multiple benefits in plants under sub-optimal conditions, including mitigation of oxidative imbalance, elicitation of osmolytes and modulation of secondary metabolism (Lucini et al., 2015). Therefore, PH-based biostimulant treatments modify plant metabolism and physiology for maximizing biomass yield under globally changing environmental conditions (Dudits et al., 2016).

In past years, significant advances were made in understanding the mode of action and in-depth characterization of biostimulants through combining omics-based methodological approaches (Rouphael et al., 2018). It was clearly demonstrated that by combining multiple omics technologies, including the high-throughput phenotyping, new functional perspectives in the biostimulant field are emerging, allowing for the discovery, evaluation, and accelerated development of innovative biostimulants (Povero et al., 2016; Bulgari et al., 2017; Rouphael and Colla, 2018; Ugena et al., 2018; Briglia et al., 2019; Dalal et al., 2019; Paul et al., 2019a).

Precise and accurate assessment of the variation in plant morpho-physiological traits over time is crucial for unraveling and quantifying the biostimulant activity of different substances. Image-based automated plant phenotyping techniques increase both the speed and the accuracy of measurements (Rouphael et al., 2018). Plant phenotyping platforms are automated systems normally operating in a fully-controlled growing chamber or in semi-controlled conditions such as greenhouses. Different sensors can be implemented into the plant phenotyping platform, allowing the user to monitor simultaneously multiple morpho-physiological plant traits in a non-destructive way. Additionally, the high number of variants and the possibility of repeated measurements from the same individuals in different phases of their growth enable the user to compare the plant development under different growth conditions and treatments, at the same time reducing costs and human labor, thus speeding up the process (Rouphael et al., 2018). As demonstrated previously by Ugena et al. (2018), multi-trait high-throughput screening (MTHTS) based on the semi-automated analysis of Arabidopsis seedlings growth provides a powerful tool for fast and large-scale discovery of new potential biostimulants, including characterization of their mode of action under optimal and stress conditions. The objective of the experiment was to use a multi-trait high-throughput screening approach based on simple RGB imaging and combined with untargeted metabolomics to screen and elucidate the mode of action/mechanisms of protein hydrolysates in Arabidopsis plants grown in optimal and in salt-stress conditions.

## Materials and Methods

### Characterization of the Protein Hydrolysates Tested

Eleven PHs were tested in the trial. Three of them were commercial products obtained by thermal-chemical hydrolysis of animal-derived proteins [Siapton^®^ (I) commercialized by Sumitomo Chemical Italia S.r.l., Milano, Italy] or enzymatic hydrolysis of vegetal-derived proteins [Trainer^®^ (D), and Vegamin^®^ (H) commercialized by Hello Nature Inc. (former Italpollina), Anderson, IN, United States]. The other eight PHs were obtained from vegetal proteins by enzymatic hydrolysis as described previously (Colantoni et al., 2017; Ceccarelli et al., 2021). Plant biomass from *Fabaceae* (A, G, O), *Malvaceae* (C), *Brassicaceae* (F), *Solanaceae* (B), and *Graminaceae* (E, P) were used as protein sources for the other eight PHs. For chemical characterization, the total C and N were determined in triplicate through an elemental analyzer (Elemental vario MAX CN, Langenselbold, Germany). Thereafter, the different PH were twofold diluted in methanol, filtered through a 0.2 membrane, and then the phytochemical profile characterized by mass spectrometry as reported by Senizza et al. (2020).

### Plant Material and Growing Conditions

*Arabidopsis thaliana* (accession Col-0) seeds were sterilized and sown as described by De Diego et al. (2017) in Murashige-Skoog (MS) medium (Murashige and Skoog, 1962) (pH 5.7) using 0.6% Phytagel (Sigma–Aldrich, Germany) as a gelling agent. To investigate the effect of biostimulants on the growth of *Arabidopsis* plantlets, the eleven PHs were dissolved in demineralized water and added to the growing media for seed priming at concentrations of 0.001, 0.01, or 0.1 μl ml^–1^. The plates containing the different media and the seeds were maintained at 4°C for 3 days and then transferred into a growth cabinet to maintain temperature and humidity setpoints (22°C, 55% RH, 16/8 h light/dark photoperiod with an irradiance of 120 μmol photons of PAR m^–2^ s^–1^).

**FIGURE 1:**
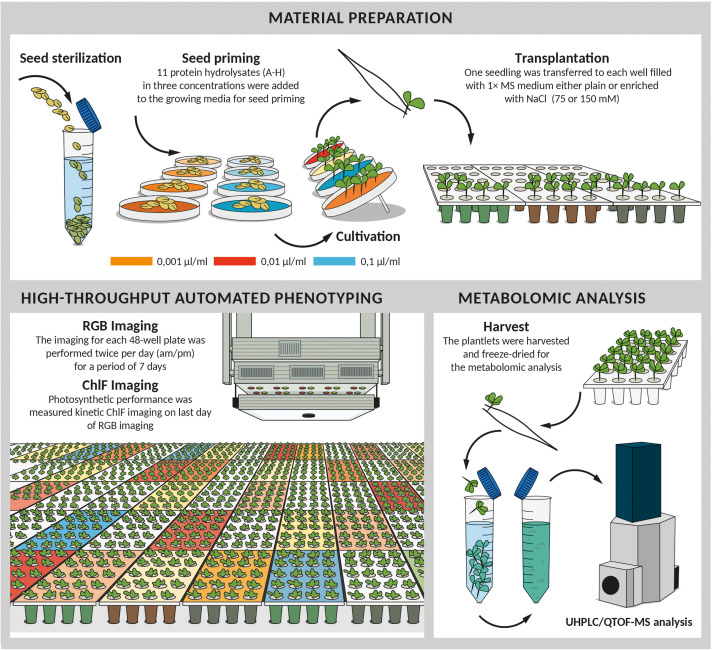
Scheme of experimental protocol for high-throughput screening of biostimulant impact on *Arabidopsis* growth in control and salinity conditions. After sterilization seeds were germinated in 0.5 × MS mixed with 11 different protein hydrolysates at three concentrations (0.001, 0.01, and 0.1 μl/ml). 4 days after cold stratification, seedlings of similar developmental stage were transplanted into 48-well plates with fresh MS medium either simple or supplemented with NaCl (75 mM or 150 mM). Plates were placed for 7 days to the cultivation chamber with XYZ PlantScreen^TM^ System used for daily (am and pm) automatic RGB imaging and growth analysis. At the end of the phenotyping period, the plates were used for the measurement of the chlorophyll fluorescence. Following the last measurement, the plantlets treated with the best-performing biostimulants including the controls were harvested, freeze-dried and used for subsequent metabolomic analysis.

Three days after germination, the seedlings were transferred into 48-well plates filled with 1 × MS medium, either plain or enriched with NaCl for two salinity levels (75 and 150 mM NaCl) as described by Ugena et al. (2018). A total of 96 seedlings (two plates) per variant as biological replicates were used. The protocol schematized in Figure 1 describes the experimental workflow.

### High-Throughput Automated Phenotyping

The plates were then transferred to the OloPhen platform (CRH Olomouc, Czechia). A generalized randomized block design was used for the random positioning of the plates in a cabinet equipped with the PlantScreen^TM^ XYZ system. The growth conditions were set to a regime of 22°C/20°C, 60% RH, and a 16/8 h light/dark cycle., while irradiance was set to 120 μmol photons of PAR m^–2^ s^–1^ (De Diego et al., 2017). Imaging was carried out twice per day (at 10:00 a.m. and 4 p.m.) for a period of 7 days (De Diego et al., 2017).

### RGB Imaging

RGB images from each plate were automatically stored in PNG format by PlantScreen^TM^ XYZ (resolution 2,500 pixels × 2,000 pixels) and analyzed using an in-house software implemented in MatLab R2015 (De Diego et al., 2017). The total number of green pixels was used to assess the total green area for each well of the plate, further referred to as the projected shoot area. The daily pictures of the single 48-well plates were then used to monitor the increase in the rosette area throughout the whole period. The Relative Growth Rate was calculated using the following formula:

$$\,\mathrm{RGR}=\frac{\ln\,\left(\mathrm{projected}\,\mathrm{shoot}\,\mathrm{area}\right)\,t_{i}-\ln\,\left(\mathrm{projected}\,\mathrm{shoot}\,\mathrm{area}\right)\,t_{i-1}}{t_{i}-t_{i-1}}$$

where *t* is the time, expressed in days.

The value of the projected rosette area from the last day of imaging was lastly used for the calculation of the Coefficient of Variance, which provides information about the size homogeneity of the seedlings on the final day of the experiment for all the treatments at all the growth conditions tested.

For the salt stress variants, a fourth growth-related parameter was introduced: Survival Rate, representing the percentage of seedlings per plate still alive on the last day of phenotyping. A seedling was considered alive if at least 100 green pixels could be detected in the corresponding well (De Diego et al., 2017).

### Chlorophyll Fluorescence Measurement

After the last RGB measurement (day 7, 10:00 a.m.), the plates were taken out of the OloPhen platform, and the perforated transparent foils were removed from each plate. Six plates at a time were randomly put onto a customized blue tray to perform kinetic chlorophyll fluorescence (ChlF) measurements of each plate, using FluorCam FC-800MF pulse amplitude modulated (PAM) chlorophyll fluorometer (Photon Systems Instruments, Drásov, Czechia) incorporated into a PlantScreen^TM^ Conveyor System. After a 15 min dark-adaptation period in the adaptation tunnel, the trays were automatically transported by the conveyor belt to the ChlF imaging light-isolated cabinet. The changes of the photosynthesis-related parameters in Arabidopsis seedlings were measured at different photon irradiances using the light curve protocol (Henley, 1993; Rascher et al., 2000). The light curve protocol with four actinic light irradiances (cool-white actinic light at 95, 210, 320, and 440 μmol m^–2^s^–1^) was used as described in Awlia et al. (2016) with a duration of 60 s, to quantify the photosynthetic efficiency. Fluorescence data were elaborated by the FluorCam7 Software (Photon Systems Instruments, Drásov, Czechia) as described by Tschiersch et al. (2017). Automation of plant masks for the single plantlets was difficult because of their small dimensions and the feeble or absent fluorescence emitted by dying or dead seedlings, especially in severe salt stress conditions. Thus, plant masks were drawn manually, using the manual image segmentation in Fluorcam7, whereas background subtraction and calculations were performed automatically. The basic ChlF parameters were derived from fluorescence transient states (i.e., *F*_*o*_, *F*_*m*_, *F*_*m*_′, *F*_*t*_, *F*_*v*_, and *F*_*p*_) and used to calculate plant photosynthetic performance parameters (*F*_*v*_/*F*_*m*_, *F*_*v*_′/*F*_*m*_′, NPQ and ΦPSII).

### Untargeted Metabolomic Analysis

Arabidopsis rosettes were freeze-dried at harvest the material from controls and primed with the best-performing substances was then used for metabolomics as described by Senizza et al. (2020). In brief, samples (10 mg) were extracted in 2 ml of a methanol-water (80:20, v/v) mixture using ultrasounds (Fisher Scientific model FB120, Pittsburgh, PA, United States) at 80% amplitude. After that, the extracts were filtered through a 0.22 μm membrane and plant metabolites analyzed by liquid chromatography quadrupole-time-of-flight mass spectrometry (UHPLC/QTOF) (Lucini et al., 2018). In summary, positive polarity and SCAN mode (100–1,000 m/z range) at 30,000 FWHM were used. Chromatography used a water and methanol binary elution mixture (from 5 to 90% methanol, 35 min run time) flowing at 220 μl min^–1^ and an Agilent Zorbax Eclipse-plus column (75 mm × 2.1 mm i.d., 1.8 μm). The software Profinder B.07 (Agilent Technologies) was used for features deconvolution, alignments and the following annotations using accurate mass, isotope spacing and isotope ratio (Rouphael et al., 2016). The reference database was PlantCyc 9.6 (Plant Metabolic Network^1^). The annotation process corresponded to Level 2 (putatively annotated compounds) of the COSMOS Metabolomics Standards Initiative (Salek et al., 2015). Compounds were finally filtered to only retain those present in 100% of replicates (*N* = 4) within at least one treatment.

### Data Analysis

One-way analysis of variance (ANOVA) with *post hoc* Tukey’s Honest Significant Difference (HSD) test (*p* < 0.05) was used for statistical differences in phenotyping data, using the MVApp application (mmjulkowska/MVApp: MVApp.pre- release_v2.0; Julkowska et al., 2019). Correlation matrices and the significance were also performed in RStudio (Version 1.1.463 – © 2009–2018 RStudio, Inc.) using the packages *factoextra, FactoMineR*, and *corrplot*.

Data from metabolomics were interpreted in Mass Profiler Professional B.12.06, (Agilent Technologies) as reported by Lucini et al. (2018). Log2 transformation and normalization at the 75th percentile were carried out prior to naive elaboration through unsupervised hierarchical cluster analysis (HCA – Wards agglomerative algorithm of the Euclidean distances). Then, Volcano Plot analysis (*p* < 0.01, fold-change >10; Bonferroni multiple testing correction) was used to identify differential metabolites in pairwise comparisons between treatments. These compounds were interpreted by the Omic Viewer Pathway Tool of PlantCyc (Stanford, CA, United States) to identify the pathways and metabolic classes elicited by the treatments (Caspi et al., 2013).

After that, OPLS-DA supervised analysis was performed in SIMCA 16 (Umetrics, Sweden) at default parameters. CV-ANOVA (*p* < 0.01) and permutation testing (*n* = 200) were used for model validation and to exclude overfitting, respectively. Fitness parameters were also calculated and Hotelling’s T2 applied to exclude outliers. Subsequently, VIP analysis was used to objectively identify the most discriminant metabolites.

## Results

### Selection and Characterization of the Protein Hydrolysates

**FIGURE 2:**
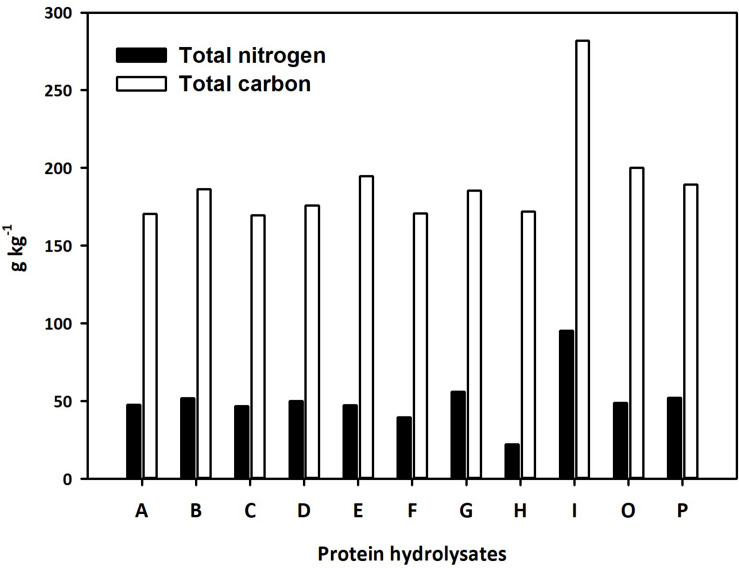
Total nitrogen and carbon content of the 11 protein hydrolysates selected for seed priming.

Eleven PHs from different natural sources were selected and used for the study. Three of the PHs were previously characterized and are commercially available products (Trainer^®^, Vegamin^®^, and Siapton^®^, here referred to as D, H, and I, respectively). The other eight PHs were obtained by enzymatic hydrolysis of plant-derived proteins and were together with the three commercial products characterized by quantitative analysis of total nitrogen and carbon. Total nitrogen in the PHs ranged between 22.2 and 95.1 g per kg of product, while total carbon content varied between 170.5 and 281.9 g per kg of product (Figure 2). The highest value of nitrogen was found in I, while H had the lowest nitrogen content. Total carbon was also highest in I, while the biostimulant A exhibited the lowest carbon concentration value. The N and C content of PHs had a positive linear correlation (*r* = 0.884^∗∗^). The untargeted analysis of the PHs revealed a broad chemical diversity that included amino acids and their derivatives, as well as other N-containing compounds (mainly alkaloids), carbohydrates (mono- to oligosaccharides), and phenylpropanoids. Relatively less polar compounds such as fatty acids and phospholipids-related compounds, carotenoids and xanthophylls, steroids and terpenoids were also represented (Supplementary Table 1). A data reduction approach based on the fold-change-based heatmap clustering was used to hierarchically describe the similarity and the difference in the whole phytochemical profile across the different PH (Supplementary Figure 1). In detail, the unsupervised clustering highlighted two main clusters, one including PH A to D and another including the products E, F, G, O, and P. The product H was distinct from these two macro-clusters, and the PH I was completely apart.

### Multi-Trait High-Throughput Screening of Arabidopsis Rosette Growth for the Characterization of the Different PHs Derived Biostimulants

The Multi-Trait High-Throughput Screening (MTHTS) described by Ugena et al. (2018) was optimized for determining the mode of action of selected PHs that were here applied as priming agents (Figure 1). The seedlings from non-primed and primed seeds with different concentrations (0.001, 0.01, or 0.1 μl ml^–1^) of PHs (Supplementary Table 2) were grown in control conditions and two intensities of salt-stress conditions. Six protein hydrolysates were evaluated in the first experimental round (A-F) and 5 in the second (G-P) round. 1st round counted 114 plates (5,472 seedlings) and the 2nd round consisted of 96 plates (4,608 plantlets), respectively. All plants were imaged by an RGB camera twice per day (at 10:00 a.m and 4 p.m.) for seven consecutive days.

Using the automated image analysis described by De Diego et al. (2017), we could quantify a variety of growth dynamics related traits such as rosette area and relative growth rate, together with homogeneity of the population (Weiner and Thomas, 1986; De Diego et al., 2017; Ugena et al., 2018).

**FIGURE 3:**
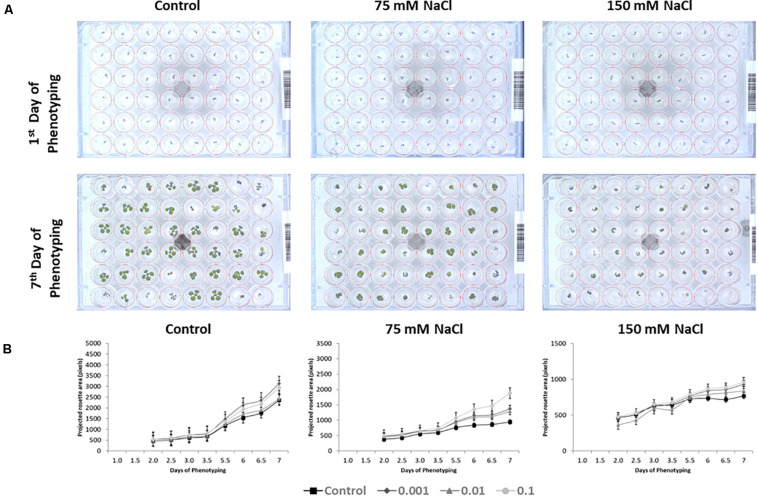
Top view RGB pictures of the 48-well plates and projected rosette area (pixels) of seedlings from seeds primed with D compound (Trainer^®^). RGB image of an individual 48-well plate at the first and the last day of the experiment, containing non-primed Arabidopsis seedlings or primed with the “D” product grown under non-saline, 75 or 150 mM NaCl conditions **(A)**. Increase in projected rosette area (pixels) throughout the 7 days of the experiment for the same seedlings primed with D product (Trainer^®^) grown under non-saline, 75 or 150 mM NaCl conditions **(B)**. The values represent the average of the 96 biological replicates per treatment, error bars represent SE.

First, we verified the reproducibility of the two rounds of the experiment, comparing the growth-related parameters of the control groups from the two rounds. Only a 2% difference between the final dimensions of the control plants in the first and the second round was observed (Rosette size of 2,362 and 2,318 pixels, respectively). This result corroborated the very high level of reproducibility of the experimental protocol used in our platform as demonstrated De Diego et al. (2017). Further, we validated the screening assay with commercial product Trainer^®^ (Hello Nature Inc., Anderson, IN, United States), here defined as substance D, that was previously characterized as growth improving substance (Figure 3) (Sestili et al., 2018).

**FIGURE 4:**
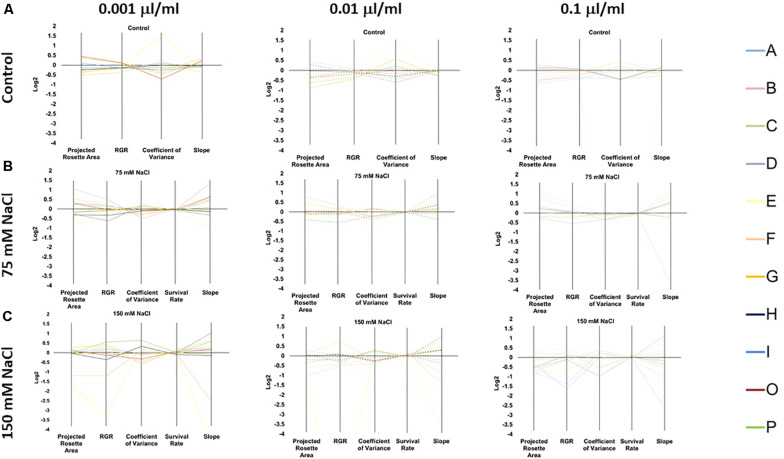
Characterization of the 11 plant biostimulants. Parallel coordinate plot of the traits (Projected Rosette Area, Relative Growth Rate, Coefficient of Variance, Survival Rate, and Slope of the Growth Curve) obtained from the Multi-trait HTS of Arabidopsis seeds primed with the PHs at three concentrations (0.001, 0.01, and 0.1 μl/ml) and grown under non-saline **(A)** 75 mM NaCl **(B)**, or 150 mM NaCl **(C)** conditions.

Overall, our phenotyping data showed that the improved growth of the Arabidopsis seedlings primed with PHs was not only product-dependent but also dose-dependent under optimal growth conditions (Figure 4A and Supplementary Table 2). The priming with all tested concentrations of C and B proved to be especially beneficial to the plant’s fitness, improving plant growth with better RGR under all growth condition, ending with a higher increase of the projected rosette area under control conditions (Figure 4A, Supplementary Table 2, and Supplementary Figure 2). In contrast, the impact on plant growth of the substances (Siapton^®^) I and O was extremely dose-dependent (Figure 4A, Supplementary Table 2 and Supplementary Figure 2). For example, when the plants were primed with I product and grown under optimal (control) conditions, the best response was observed in the highest concentration of the substance (0.1 μl ml^–1^). In contrast, O product had the best effect when the lowest dose was used as a priming agent, while the highest concentration caused the opposite effect and resulted in the reduction of the final rosette area (Figure 4A, Supplementary Table 2, and Supplementary Figure 2). As expected, O is not the only substance that proved to be growth-inhibiting and/or toxic to the plants at a very high dose. The same detrimental effect was observed in groups primed with A, B, F, G, and P. In summary, from our data, it is possible to identify the substances C and D (Trainer^®^) as the best growth promoters.

### Influence of the Protein Hydrolysates Applied as Seed Priming on Rosette Growth and Survival in Salt Stress Conditions

Thanks to the capacity of the OloPhen platform, we were able to evaluate the effect of our substances under two levels of salt stress: moderate (75 mM NaCl) and severe (150 mM NaCl). The two NaCl concentrations were selected based on the work from De Diego et al. (2017). As a main result, we observed that for the seedlings primed with PHs, independently of the origin of the substances used for the priming, the stress-induced growth inhibition was usually alleviated so in many cases bigger Arabidopsis rosettes and RGR were observed. However, the effects of the PHs on the seedlings were extremely dose-dependent and dependent on the severity of the salt stress applied.

At 75 mM NaCl, only 3 of the 11 substances (C, D, and O) significantly increased the final projected rosette area compared to the non-primed seedlings (Figure 4B, Supplementary Table 2, and Supplementary Figure 3). The beneficial effect of C, D, and O was significant over all concentrations tested, resulting in dose-independent stress alleviating action. Interestingly, C’s improving effect was more noticeable in the lowest concentration (0.001 μl ml^–1^), whereas for D in the highest concentration (0.1 μl ml^–1^). This result suggested that these two products varied in the mode of action for mitigating the negative effects of salt stress. Similarly, only the seed priming with any of the concentrations of C or D (Trainer^®^) improved the RGR values showing that only 2 of the PHs used (C and D) behaved as stress alleviators (Figure 4B, Supplementary Table 2, and Supplementary Figure 3), with higher values for 0.001 μl ml^–1^ in C and 0.1 μl ml^–1^ in D (Trainer^®^). Interestingly, E and I (Siapton^®^) had an inhibiting effect on RGR, reducing this parameter in all three concentrations.

For the salt stress variants, a third growth-related parameter was introduced; the survival rate (%) was calculated on the last day of the experiment. The survival rate of the seedlings was not seriously compromised in moderate salt stress conditions (∼100%) (Figure 4B and Supplementary Table 2).

At 150 mM NaCl, no substance caused an increase in the final area (Figure 4C, Supplementary Figure 4, and Supplementary Table 2). However, the RGR was improved by the seed priming with D and P substances. D (Trainer^®^) acted as a stress alleviator in all concentrations, especially with 0.01 μl ml^–1^ dose, whereas P substance only improved the RGR when the highest concentration (0.1 μl ml^–1^) was used. Contrarily, E and F inhibited the growth of the seedlings in all concentrations. Severe salt stress also reduced the survival rate of the seedlings, with values around 95% for unprimed plants. The seed priming with B, C, D, and O maintained higher survival rates but the effect was present in a dose-dependent manner (Supplementary Table 2); the most effective concentration for C and D (Trainer^®^) was 0.001 μl ml^–1^, while for B and O was 0.1 μl ml^–1^. Remarkably, the seeds priming with E and F at all concentrations had a reduced survival rate.

### Influence of Seed Priming With Protein Hydrolysates on Seedlings Homogeneity

Despite the two selection steps for the plant material (seed size and seedling size at the transfer moment), some variability between seedlings is always present. However, the level of variability in the population can be modified by the growth conditions and/or priming agents (De Diego et al., 2017; Ugena et al., 2018).

For that, we evaluated the effects of the priming with the different PHs on the plant-to-plant variability. The coefficient of variation (CV = standard deviation/mean) was used as a standard measurement of relative variation (Weiner and Thomas, 1986) and calculated on the last day of phenotyping, before the harvest. In control conditions, O was the only substance that improved the homogeneity of the seedlings, except when it was applied at the highest concentration. In conditions of moderate salt stress, the CV was reduced by C in the 0.001 μl ml^–1^ concentration and by E in the 0.01 μl ml^–1^ concentration compared with their respective control. In severe stress conditions, the highest variability occurred, probably because most of the seedlings stopped growing in the early phase of the experiment (Figure 4C and Supplementary Table 2). In this case, the substances B (in the 0.1 μl ml^–1^ concentration), D (Trainer^®^ at 0.001 μl ml^–1^), H (Vegamin^®^ at 0.01 μl ml^–1^), and O (0.1 μl ml^–1^) improved the uniformity of the plantlets significantly (Figure 4C and Supplementary Table 2).

### Evaluation and Classification of the Substances Through the Plant Biostimulant Characterization Index

In order to uniquely classify the 11 PHs according to their effect on seedlings as growth promoters and/or stress alleviators, we used the Plant Biostimulant Characterization (PBC) index developed by Ugena et al. (2018). This index considers the five parameters previously mentioned: Projected Rosette Area on the last day of measuring, Relative Growth Rate throughout the entire period of the experiment, coefficient of variance in the final day of the experiment, the slope of the growth curve, and the final survival rate for the variants grown under salt stress conditions. The log2 of the ratio between primed and unprimed seedlings was calculated for each of the five parameters, the concentration of the PHs (0.001, 0.01, or 0.1 μl ml^–1^) and growth conditions [optimal (control), moderate salt stress (75 mM NaCl) or severe salt stress (150 mM NaCl)], values that concur with those represented in the parallel plot (Figure 4). As example, for the A substance at 0.001 μl ml^–1^ applied to the plants growing in moderate salt stress conditions (75 mM NaCl), the log2 of the analyzed traits were: for final area [log2 (1184.25/947) = 0.3225], for RGR [log2 (0.20/0.18) = 0.1448], for CV [−log2 (53.5665071/55.38435406) = −0.0481, as it is a negative trait], for survival [log2 (95.83/100) = −0.0614] and for slope [log2 (149.1805556/106.3796296) = 0.4878]. The five values obtained were then summed up to calculate the PBC index, ending with a single numeric value that could categorize the compounds in a straight-forward way. The value obtained for the single compound, concentration and growth condition could be negative (red) or positive (blue), telling us if this specific combination was beneficial in terms of plant performance in the given conditions compared to the respective control variant (from non-primed seeds) (Table 1). Additionally, the obtained values allowed us to divide the compounds into three groups; plant growth promotor [only positive values (blue) in primed seedlings grown under control conditions], stress alleviator [only positive values (blue) in primed seedlings grown under stress conditions], or both [positive values in primed seedlings under control and stress conditions].

**TABLE 1:** Plant biostimulant characterization (PBC) index.

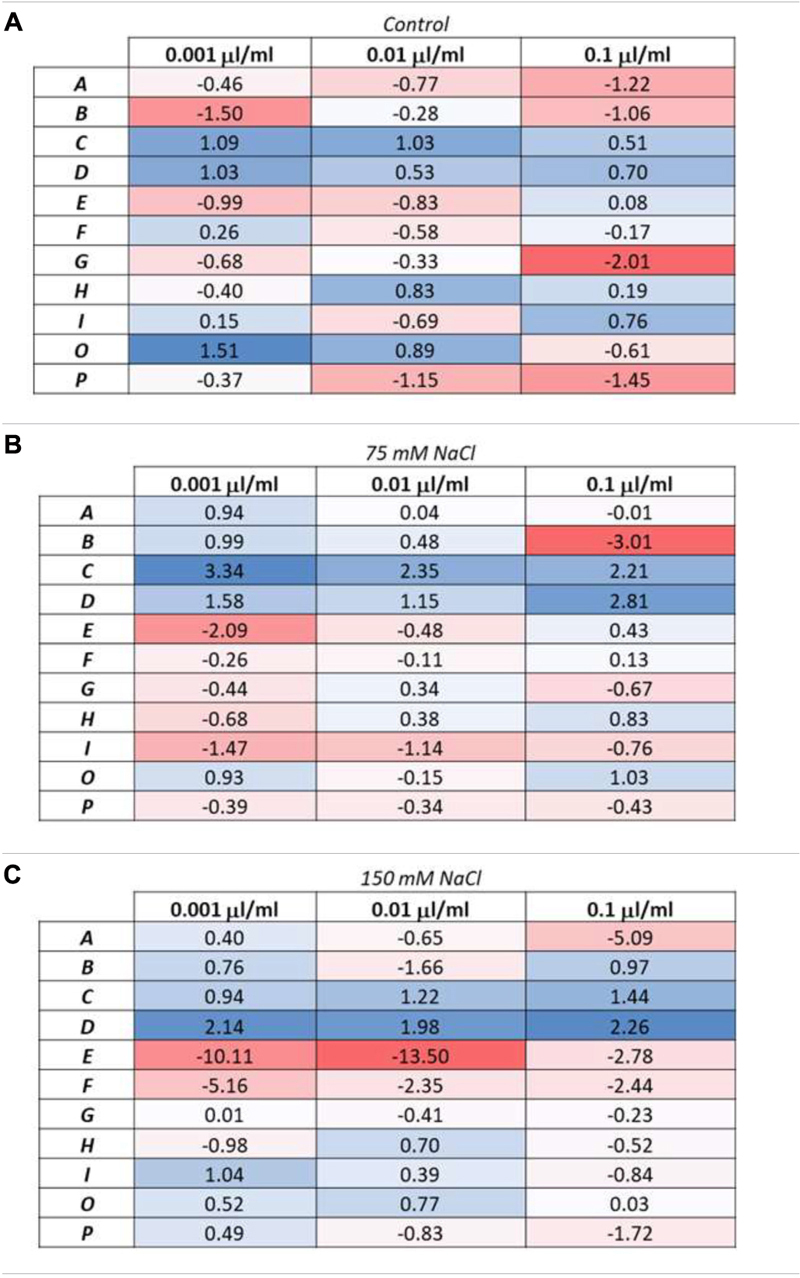

*The PBC index (related to each control) calculated as the sum of the log2 ratio between control and primed seedlings for the five main traits extracted from the RGB images (Parallel plot, Figure 4) measured under control****(A)***, *moderate****(B)***, *and severe salt stress conditions****(C)***. *Red color and blue color mean worse and better performance than the non-primed variants, respectively.*

Overall, our data clearly suggest that C and D (Trainer^®^) were the best biostimulants in all their concentrations and growth conditions, acting both as plant growth promotors and salt stress alleviators. C was especially effective as stress alleviator in the 75 mM NaCl-enriched media, with a PBC value 208% (for 0.001 μl ml^–1^), 129% (for 0.01 μl ml^–1^) and 335% (for 0.1 μl ml^–1^) higher than the non-primed seedlings. On the contrary, D (Trainer^®^) had a better stress-alleviating effect on the plants growing in 150 mM NaCl- enriched media, with a PBC value 108% (for 0.001 μl ml^–1^), 276% (for 0.01 μl ml^–1^) and 221% (for 0.1 μl ml^–1^) higher than the non-primed variant.

Some of the remaining substances proved to be effective as well, although in a concentration and growth condition-dependent manner. For example, as the PBC index shows (Table 1A), the substance O can be classified as a growth promotor when applied at the two lower concentrations. At the same time, the highest dose was even detrimental to the plantlets’ development in control conditions. The substance B, however, can be classified as a stress alleviator but when used in high doses it was inhibiting the growth when plants were grown in 75 mM NaCl-enriched media (Table 1B). E and F were the worst performing substances, inhibiting the growth of the seedlings in all concentrations and growing conditions and especially under 150 mM NaCl salinity conditions (Table 1C).

### Influence of Protein Hydrolysates on Photosynthetic Performance

To verify the effect of the priming on the photosynthetic performance of the seedlings, a range of ChlF parameters was measured using the PAM method and light curve quenching kinetics on the last day of the experiment, after the RGB imaging for all the plates was completed. A set of fluorescence parameters reflecting the photosynthetic function of PSII were calculated (Supplementary Table 3). The maximum quantum yield of photosystem II in dark-adapted (*F*_*v*_/*F*_*m*_) was used to characterize photosynthetic performance of the control and stressed seedlings (Supplementary Figure 5). *F*_*v*_/*F*_*m*_ was shown to be a robust indicator of plant stress (Rousseau et al., 2013; Wang et al., 2016; Wu et al., 2018) and especially of salt stress (Lucini et al., 2015; Simko et al., 2016; Adhikari et al., 2019). In our experiment, the value of *F*_*v*_/*F*_*m*_ was significantly reduced in the plants grown in the 150 mM NaCl-enriched media, but not under moderate salt stress (Supplementary Figure 5). Overall, the seedling’s photosynthetic efficiency belonging to the moderate stress group was not severely compromised (Supplementary Figure 5 and Supplementary Table 3). Only the seedlings primed with 0.01 μl ml^–1^ and the 0.1 μl ml^–1^ H (Vegamin^®^), or with a 0.1 μl ml^–1^ solution of A and O improved the *F*_*v*_/*F*_*m*_ under moderate stress conditions. The seed priming with the highest concentration of the substance F was even able to increase the value *F*_*v*_/*F*_*m*_ higher than the values observed in the non-primed seeds grown under optimal conditions in control conditions (Supplementary Figure 5 and Supplementary Table 3). Contrarily, B at the 0.001 ml^–1^ concentration negatively affected the photosynthetic performances of the seedlings in moderate stress conditions, reducing the *F*_*v*_/*F*_*m*_ values to those observed in the plants grown under severe salt stress (150 mM NaCl) (Supplementary Figure 5 and Supplementary Table 3).

**FIGURE 5:**
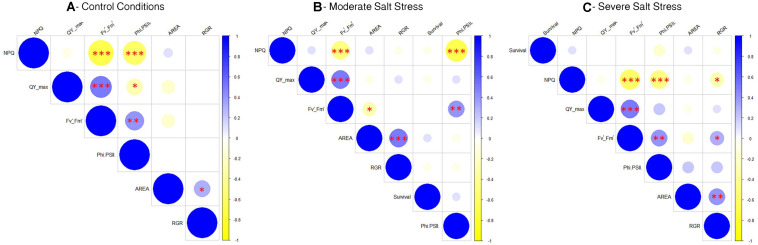
Correlation matrices comparing the growth and fluorescence related phenotyping traits in Arabidopsis seedling grown under control conditions **(A)**, moderate salt stress **(B)**, or severe salt stress **(C)**. Red asterisks mean ^∗^*p* < 0.05, ^∗∗^*p* < 0.01, ^***^p < 0.001.

Finally, to understand how the fluorescence parameters conditioned plant growth under the three different growth conditions studied, we performed three different correlation matrices using the phenotyping data per well plate (a total of 70 plates per growth condition) (Figure 5). As a result, there was not a clear correlation between the growth parameters (rosette size and RGR) with the fluorescence parameters under control and moderate salt stress conditions (Figures 5A,B). However, under severe stress conditions the RGR was positively correlated with higher *F*_*v*_′/*F*_*m*_′ (^∗^*p* < 0.05) and negatively with NPQ (^∗^*p* < 0.05) (Figure 5C), showing that under severe salt stress a higher photosynthetic efficiency related with the RGR and hence, the plant growth and final rosette size.

### Metabolomics Insights Into the Mode of Action of Selected Protein Hydrolysates

Once the best performing substances were identified according to the PBC index (C and D), we carried out a non-targeted metabolomic analysis based on UHPLC-ESI/QTOF-MS. The priming seedlings from these treatments, together with their respective controls were collected at the end of the phenotyping experiment. The metabolic analysis also included the three studied growth conditions [optimal growth conditions (control), and moderate (75 mM NaCl) or severe (150 mM NaCl) salt stress], in which seedlings from non-primed or primed seeds with the substance C (*Malvaceae*-derived PH) or D (Trainer^®^) were compared. The lowest concentration (0.001 μl/l) was selected for the analysis of Arabidopsis seedlings grown under control and moderate stress conditions for two main reasons; this concentration presented the highest PCB index values in both substances (Table 1) and because the use of lower concentration has economic benefits. However, under severe salt stress the highest concentration 0.1 μl/l of D and C was analyzed because it showed the best performance in the phenotyping data (Table 1). The whole list of metabolites annotated, together with individual abundances and composite mass spectra, is provided as Supplementary Table 4.

**FIGURE 6:**
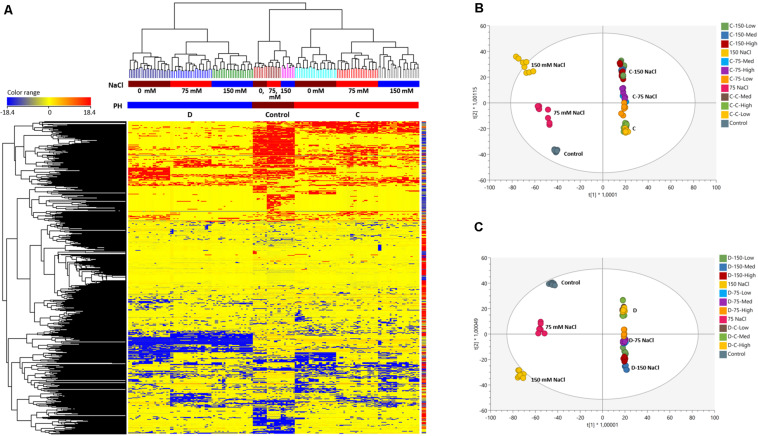
Fold-change based unsupervised hierarchical cluster analysis (Euclidean distance) carried out from metabolomic profile of plants treated either with the biostimulant C or D, at different salinity levels **(A)**; OPLS-DA (Orthogonal Projections to Latent Structures Discriminant Analysis) supervised modeling of metabolomic profile in plants at different salinity levels and treated with the OPLS-DA of the two best performing protein hydrolysates, C **(B)** and D **(C)**.

The unsupervised hierarchical clustering indicated different metabolic profiles when comparing non-primed or primed seedlings, as thereafter confirmed by the supervised OPLS-DA modeling (Figure 6). The clustering built from the fold-change based heatmap (Figure 6A) highlighted two main clusters: a first including the seedlings primed with the D substance under the three tested growth conditions, and a second cluster with the non-primed seedlings and those primed with the C substance. This last cluster was also divided into two subclusters that separated the non-primed seeds from the primed ones with substance C, independent of the growing conditions. These results indicated that the main clustering factor was the type of priming agent used.

To corroborate these results, we performed an OPLS-DA supervised multivariate analysis, in which the two substances were independently compared to the non-primed seeds (controls) under the three tested growth conditions (Figures 6B,C). In both analyses, the results provided a score plot in agreement with hierarchical clustering, showing that the priming agent is the principal factor separating the samples followed by the growth conditions (Figures 6B,C). The investigation of the most discriminant compounds in both OPLS-DA models (i.e., variables of importance in projection—VIP analysis) was then carried out. The Supplementary Table 5 includes two columns (one for the substance C and another for the substance D) reporting the discriminant metabolites identified (VIP score >1.3). Overall, from the comparison between non-primed seeds and the seeds primed with the C or D substance (Sheets- VIPs markers C or VIPs markets D) 97 and 127 compounds were identified, respectively. Due to this different metabolic profiling between the plants from seeds primed with C or D substance, we also carried out an additional OPLS-DA and identified the most discriminant compounds that differed between these two treatments. As an outcome, a total of 253 compounds were identified (Supplementary Table 6), confirming that C and D substances affected in different ways the seeds and hence the plant growth. For example, only the D-primed seedlings increased compounds such as β-solanine, guaiacol or plant hormones-related compounds such as gibberellin 34, the brassinosteroids 6-deoxo-24-epicathasterone and campest-5-en-3-one, the sugar maltose or some flavones such as baicalin and 7-hydroxyflavone, among others (Supplementary Table 6). However, C but not D increased certain sesquiterpenoids such as curcuquinone or the main precursor for the synthesis of the aromatic amino acids, shikimate, relevant pathway controlling plant growth and development (Tzin and Galili, 2010), or the metabolite *meso*-diaminopimelate, substrate for the synthesis of L-lysine (Crowther et al., 2019) (Supplementary Table 5).

**FIGURE 7:**
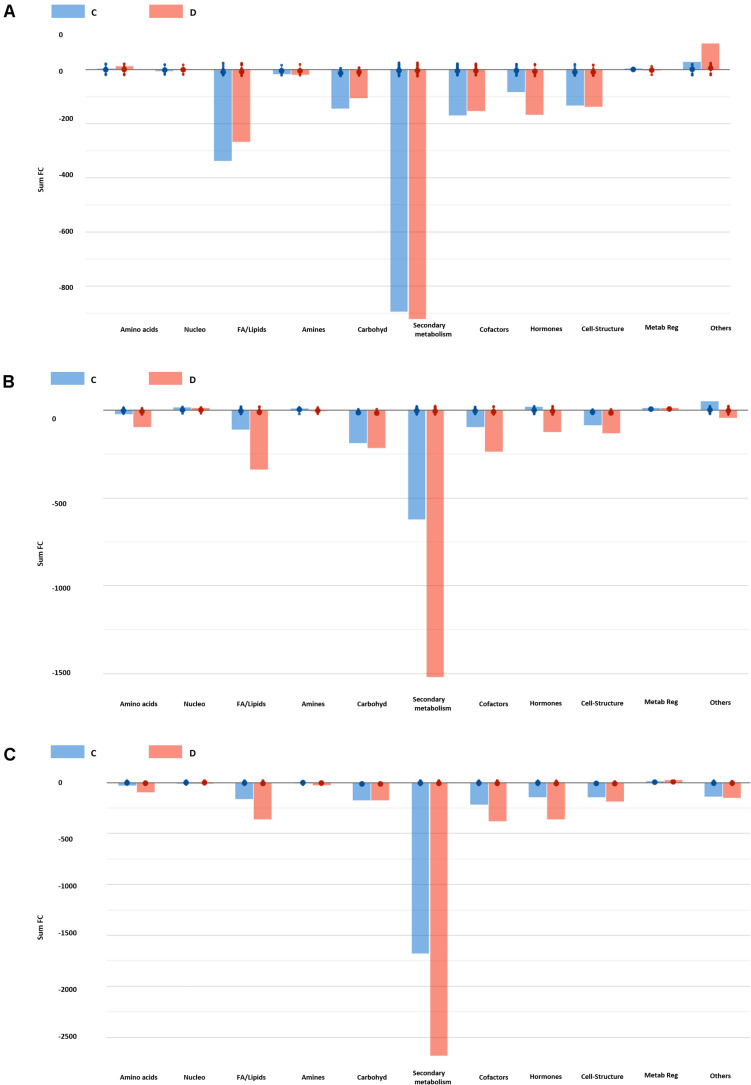
Biosynthetic processes affected by the two best performing protein hydrolysates at 0 **(A)**, 75 **(B)**, and 150 mM NaCl **(C)**. Differential metabolites (Volcano Plot analysis, *n* = 8) and their fold-change (FC) values were elaborated using the Omic Viewer Dashboard of the PlantCyc pathway Tool software (www.pmn.plantcyc.com). Within each class, large and small dots represent the average (mean) and individual logFC, respectively.

To go further with the study of the mode of action, we inferred the biochemical processes that these two substances are activating in the *Arabidopsis* seedlings to modulate plant growth and promote stress alleviation. To this aim, the discriminating compounds were compared in each growth condition by Volcano Plot analysis (Figure 7 and Supplementary Table 7). First of all, the different compounds were grouped in functional classes; synthesis of amino acids, nucleotides, carbohydrates, fatty acids or lipids, hormones, cofactor synthesis with the metabolites related to secondary metabolism being the most represented in all the growth conditions, especially in the case of Trainer^®^ (D) (Figure 7). Secondly, the compounds that differ the most (opposite behavior as in one up accumulated and in another one without changes or down accumulated) between the two PHs were identified (Supplementary Table 6). As an example, when the plants primed with D substance were grown under moderate salt stress (75 mM NaCl), secondary metabolites such as flavonoids and terpenes decreased.

A similar profile was observed when plants were grown under severe salt stress conditions (150 mM NaCl) (Figure 7C). However, in this case many derivate forms of plant hormones such as benzyladenine-7-glucoside, 16,17-dihydro-16α-17-dihydroxy gibberellin 12 and methylgibberellin 4, the IAA-derivate 4-(indol-3-yl)butanoyl-β-D-glucose or the brassinosteroid castasterone were highly reduced in the seedlings primed with D substance but not with C, compared to the plants coming from non-primed seeds. All together, it is clear that being both PH products, including when they are from the same type of botanical material but not the same family, their application to the plants affect different metabolic pathways, including the phytohormone balance, that finally condition the plant response to the environment in which is grown.

## Discussion

Sustainable approaches able to promote plant growth and enhance crop productivity represent a priority in modern agriculture (Xu and Geelen, 2018). Protein hydrolysates, as natural products mainly deriving from agricultural waste and able to reduce dependency on chemical fertilizers, are therefore of great interest. However, due to the diverse origins of the biostimulants, their manufacturers require fast and efficient tools for identifying and characterizing new functional biostimulants and to identify their mode of action (Ugena et al., 2018). In the last years, platforms for high-throughput automated phenotyping have been frequently used for fast and highly reproducible screenings of the effects of potential biostimulants on growth-related traits of plants, both in control and stress conditions (Rahaman et al., 2015; De Diego et al., 2017; Ugena et al., 2018; Paul et al., 2019a,b). However, most platforms are limited in their capacity of measuring a large number of individuals (or variants) at the same time. In contrast, the comparison between plants primed with different doses of biostimulants and growing in diverse stress severities is fundamental to prove the effectiveness of the substances as biostimulants and elucidate their mode of action. The biostimulant activity of a product, in fact, is strongly dependent on the severity of the stress applied to the plant (Bulgari et al., 2019) as well as on the time of exposure; therefore, the beneficial effects of a substance can vary with the concentration and time of exposure of the plants to the stress (Colla et al., 2010). Transferring to *in vitro* conditions using a model plant such as *Arabidopsis* allows increasing the number of treatments and replicates (De Diego et al., 2017). Starting from these premises, we followed the same protocol described by Ugena et al. (2018). The effects of potential biostimulant substances were tested on *Arabidopsis* seedlings grown under optimal conditions and salt stress in two different intensities (75 mM and 150 mM NaCl). However, instead of using single compounds such as polyamines, we tested the effects of 11 complex products based on protein hydrolysates from different natural origins, applied in three different concentrations (0.001, 0.01, and 0.1 μl ml^–1^) as seed priming agents. Priming induces preliminary germination (Jisha et al., 2012; Paparella et al., 2015), enhances synchronized germination, promotes plant growth (Bryksová et al., 2020) and can elicit resilience to stressors (Conrath, 2011; Paparella et al., 2015). Priming can improve seed performance, ensure higher uniformity among the seeds, result in faster and better. Priming finds application particularly in vegetables like carrot, onion, celery, lettuce, endive, pepper, and tomato (Paparella et al., 2015). This is why in our study the seed priming with PHs-based substances was used instead of mixing them into the media, so the amount of the substances used for the priming is highly reduced saving product and costs, and of course reducing the potential toxicity of the high dosages. As corroboration, we could see that the seed priming with the high dosages of some PHs-based substances inhibited plant growth (Table 1 and Figure 4) but did not kill the plants as happened in previous studies in which the substances were applied to the growth media (data not shown).

Simple RGB daily pictures were able to provide us with plenty of information related to the plants’ growth and fitness using the MTHTS approach: starting from the mere dimensions of the plants, we could calculate the slope of the growth curve, the RGR, the Coefficient of Variance and the Survival Rate in salt stress conditions. Exactly as described by Ugena et al. (2018), the phenotyping traits were used to calculate the Plant Biostimulant Characterization (PBC) index, which ended with a single number making easier the characterization of each biostimulant according to their mode of action: growth promotor/inhibitor and/or stress alleviator. Thus, the PBC index showed that the effects of the substances on plants was not only dependent of the PH substance tested but also dose dependent. For most of the substances, the highest concentration (0.1 μl ml^–1^) was not beneficial or reduced plant growth (Table 1 and Figure 4). It is known that PHs contain carbohydrates, amino acids, and lipids that may improve crop fitness, acting as plant growth regulators (growth promoters) in the absence of any external stress, due to the presence of bioactive peptides (Colla et al., 2014, 2015) with a range of phytohormone-like activities (Ito et al., 2006; Kondo et al., 2006). PHs may as well increase plant tolerance to abiotic stresses (Van Oosten et al., 2017) because certain amino acids affect the ion fluxes across membranes, most having a positive effect on reducing NaCl-induced potassium efflux (Cuin and Shabala, 2007). However, when PHs based substances are applied to the plants at high dosages an excess of free amino acids or phenols can have the opposite effect and induce growth retardation (Cerdán et al., 2009; Muscolo et al., 2013; Ertani et al., 2018), explaining the inhibitory effect observed in some of the variants. Only the substances C and D (Trainer^®^) improved plant growth under control and stress conditions, including when they were applied in high concentration, with better results in the case of D (Trainer^®^), our positive control. In this regard, Trainer^®^ has been demonstrated to improve the growth of many crop species and to mitigate the deleterious effects of salt stress (Colla et al., 2014; Lucini et al., 2015; Rouphael et al., 2017; Di Mola et al., 2019; Luziatelli et al., 2019; Paul et al., 2019b). Altogether, we showed that the MTHTS of Arabidopsis rosette growth is an advantageous and fast approach to test new biostimulants under a wide range of concentrations and growth conditions. Besides, our results are comparable with those obtained in other interesting plant species including crops with agronomical interest, confirming the biological translation of the results obtained in Arabidopsis to them. The PHs-derived biostimulants C and D have in common the vegetal origin but differ in the plant family from which they are produced (*Malvaceae* and *Fabaceae*, respectively).

At the end of the experiment, chlorophyll fluorescence measurement of all the plants have also been performed and the light curve protocol (Henley, 1993; Rascher et al., 2000) was used as it was proven to be especially effective in providing detailed information on plant adaptation to adverse conditions (Brestic and Zivcak, 2013; Awlia et al., 2016). As a result, we observed that the maximum quantum yield of PSII photochemistry in the dark-adapted state (*F*_*v*_/*F*_*m*_) was reduced in salt stress conditions, especially in the 150 mM NaCl-enriched media. This is coherent with previous works (Baker and Rosenqvist, 2004; Awlia et al., 2016), where *F*_*v*_/*F*_*m*_ proved to be a robust parameter, being affected only under severe stress. Additionally, the seed priming with some PHs based substances at certain concentrations also improved the *F*_*v*_/*F*_*m*_ under optimal and salt stress conditions (Supplementary Figure 5 and Supplementary Table 3). This is in agreement with previous experiments, in which the use of plant-derived PHs promoted photosynthetic efficiency and increased the accumulation of photosynthetic pigments (Yakhin et al., 2017). However, this effect was not very remarkable in the case of the best performing PH (D). A possible explanation is that this product did not influence the light phase of the photosynthesis (fluorescence parameters) but could increase the dark phase of the photosynthesis and hence, the efficiency of the plant, as has been described previously in PHs treated lettuce (Xu and Mou, 2017).

Another explanation for this result can relate to the broad metabolic reprogramming induced by PHs. For example, the seedlings primed with D substance accumulated higher levels of maltose compared to the controls. Maltose is a soluble sugar and the major starch-degrading product (Thalmann and Santelia, 2017). Starch degradation (a common plant stress response) is the main mechanism D- primed plants used, resulting in accumulating certain soluble sugars, especially maltose (Supplementary Table 5). As corroboration of the beneficial maltose accumulation, its exogenous application in wheat plants improved plant growth, yield and some biochemical components when grown under drought conditions (Ibrahim and Abdellatif, 2016). The Arabidopsis seedlings primed with D substance also displayed lower levels of flavonoids and terpenoids. These compounds are mainly accumulated in plants under stress condition resulting in reactive oxygen species (ROS) production (D’Amelia et al., 2018; Sharma et al., 2019a). Altogether, we suggest that the reduced presence of flavonoids and terpenoids pointed to the D-primed seedlings as healthier plants with lower levels of ROS that allow the plants to grow better. Finally, recent studies have shown strong crosstalk between flavonoids and some plant growth regulators such auxins and cytokinins, controlling biological processes such as nodulation in *Medicago truncatula* and, hence, plant growth (Ng et al., 2015). In this regard, the D-treated plants showed a clear reduction in many products of degradation or conjugation (mainly related to inactivation) of cytokinins, auxins, and brassinosteroids. Thus, they could maintain the levels of the active phytohormone forms to preserve the general homeostasis of the plants. In this regard, both the activation and inactivation of cytokinin degradation genes have been mentioned to give plant stress tolerance (Vojta et al., 2016; Prerostova et al., 2018). In Arabidopsis, for example, the inducible 35S:CKX plants were approximately half those of WT plants under well-watered conditions, their rosette growth rates were actually more sensitive to soil drying, and they recovered more slowly after re-watering (Prerostova et al., 2018). These results are in accordance with the better growth of the D-treated Arabidopsis seedlings and the reduced benzyladenine-7-glucoside levels. Finally, these seedlings also accumulated brassinosteroid precursors such as 6-deoxo-24-epicathasterone and campest-5-en-3-one and reduced the formation of castasterone. Brassinosteroids (BRs) are a category of plant steroid hormones having multiple roles in plant growth, development, and stress responses (Ahammed et al., 2020). In fact, the accumulation of castasterone has been related to plant stress response and detoxification under metal and pesticide stress (Sharma et al., 2019b; Ahammed et al., 2020). Interestingly, brassinosteroids have been reported to modulate plant growth and stress management, including under saline conditions (Vidya Vardhini, 2017). This suggests a lower level of castasterone indicated that the plants experienced new homeostasis in which the effect or toxicity induced by salt stress is reduced. Our findings indicate that this modulation of brassinosteroids might be the consequence of an improved resilience toward salinity induced by the biostimulants in our plants.

## Conclusion

The present study presented a complex pipe-plan to select and understand the mechanism of action of 11 PHs-substances used as priming agents. The results demonstrated that the high-throughput phenotyping approach, such as MTHTS of Arabidopsis rosette, is a valuable tool to compare a high number of biostimulants at different concentrations in plants grown under different conditions (with and without stress). This approach has proven to be able to accelerate the selection of the best performing substances in a highly effective manner. Besides, the obtained results corroborated the biological translation from Arabidopsis to other crops with agronomical interest. Additionally, the combination of the phenomics with untargeted metabolic analysis revealed that the priming with the best-performing substance modifies the plant homeostasis thus promoting growth and allowing a higher survival by reducing the oxidative damages induced by the stress and by regulating the crosstalk between different plant hormones. Finally, this approach can help to accelerate the selection and characterization of new biostimulants that make the plants more efficient and more resistant to stress. Further studies will be performed using model crops to go further in the understanding of the mode of action of PHs based biostimulants.

## Data Availability Statement

The original contributions generated for this study are included in the article/Supplementary Material, further inquiries can be directed to the corresponding authors.

## Author Contributions

YR, LL, and GC prepared and selected the protein hydrolysates. MS, ND, LS, and KP designed the phenotyping experiments. MS and LU performed the phenotyping experiments. MS and ND performed the image processing and image-based data analysis. LL, BM-M, and LZ carried out the untargeted metabolomics. LL, BM-M, and ND analyzed the metabolomic results. GC and KP coordinated the whole project. All authors discussed the results and contributed to writing the manuscript.

## Conflict of Interest

KP was employed by the company Photon Systems Instruments, spol. s r.o. The remaining authors declare that the research was conducted in the absence of any commercial or financial relationships that could be construed as a potential conflict of interest.

## Footnotes

1 http://www.plantcyc.org

## Supplementary Material

Supplementary Figure 1 Hierarchical cluster analysis conducted on the phytochemical composition of the different PHs; a fold-change heat map was done, and Euclidean distance used for clustering.

Supplementary Figure 2 Growth of the plants in control conditions following the priming with the set of protein hydrolysates. Projected rosette area (pixels) of Arabidopsis seedlings primed with the 11 protein hydrolysates **(A–P)** at three concentrations (0.001, 0.01, and 0.1 μl/ml) and grown for 7 days in 48-well plates under control conditions. Rosette area was extracted from RGB images acquired twice a day (am and pm) over the period of 1 week. Values represent the average of the 96 biological replicates per treatment, bars represent SE.

Supplementary Figure 3 Growth of the plants in moderate salt stress conditions following the priming with the set of protein hydrolysates. Projected rosette area (pixels) of Arabidopsis seedlings primed with the 11 protein hydrolysates **(A–P)** at three concentrations (0.001, 0.01, and 0.1 ml/ml) and grown for 7 days in 48-well plates under moderate (75 mM NaCl) salt stress conditions. Rosette area was extracted from RGB images acquired twice a day (am and pm) over the period of 1 week. Values represent the average of the 96 biological replicates per treatment, bars represent SE.

Supplementary Figure 4 Growth of the plants in severe salt stress conditions following the priming with the set of protein hydrolysates. Projected rosette area (pixels) of Arabidopsis seedlings primed with the 11 protein hydrolysates **(A–P)** at three concentrations (0.001, 0.01, and 0.1 ml/ml) and grown for 7 days in 48-well plates under severe (150 mM NaCl) salt stress conditions. Rosette area was extracted from RGB images acquired twice a day (am and pm) over the period of 1 week. Values represent the average of the 96 biological replicates per treatment, bars represent SE.

Supplementary Figure 5 Maximum quantum yield of PSII photochemistry in the dark-adapted state (*F*_*v*_/*F*_*m*_) of the Arabidopsis seedlings. Graphs show the maximum quantum yield of the plantlets after 7 days of in control, moderate (75 mM NaCl), and severe (150 mM NaCl) salt stress conditions. Seedlings were primed with the 11 protein hydrolysates at three concentrations (0.001, 0.01, and 0.1 ml/ml). Values represent the average of the 96 biological replicates per treatment, bars represent SE. Different letters are used to indicate the significant differences between the treatments (different PH’s and control treatment) using *post hoc* Tukey’s test (*p* < 0.05).

Supplementary Table 1 Untargeted phytochemical composition in the tested PHs; individual abundances (as provided from UHPLC/QTOF metabolomics) are also provided.

Supplementary Table 2 Growth-related parameters extracted from the RGB images. Values for the Slope of the growth curve, projected rosette area (pixels) in the last day of measurement, RGR (pixel pixel-1 day-1) for the entire period of the experiment and survival rate (%) estimated at the last day of the trial. The values displayed correspond to Arabidopsis seedlings from non-primed seeds or primed with 11 different PHs at 3 concentrations (0.001, 0.01, and 0.1 ml/ml) grown under non-saline **(A)**, 75 **(B)**, and 150 mM NaCl conditions **(C)**. Different letters are used to indicate the significant differences between the treatments (different PH’s and control treatment) using *post hoc* Tukey’s test (*p* < 0.05).

Supplementary Table 3 Fluorescence-related parameters. Values of the maximum quantum yield of PSII photochemistry in the dark-adapted state (*F*_*v*_/*F*_*m*_), the maximum quantum efficiency in light-adapted state (*F*_*v*_′/*F*_*m*_′), the steady-state non-photochemical quenching (NPQ) and the steady-state operating efficiency of PSII in the light (ΦPSII) in the last day of the trial of the experiment. The values displayed correspond to plantlets treated with all the 11 PHs, in the 3 concentrations (0.001, 0.01, and 0.1 ml/ml), in non-saline **(A)**, 75 **(B)**, and 150 mM NaCl conditions **(C)**. Different letters are used to indicate the significant differences between the treatments (different PH’s and control treatment) using *post hoc* Tukey’s test (p < 0.05).

Supplementary Table 4 Whole list of metabolites annotated through untargeted metabolomics in plants, either treated or not with the biostimulants, at different salinity levels. Metabolites are reported with individual abundances, PlantCyc annotation, composite mass spectrum (mass-abundance combinations) and retention time.

Supplementary Table 5 OPLS-DA modeling VIP markers are also provided with score and standard error for protein hydrolysate C and D separately.

Supplementary Table 6 VIP markers (with individual score and standard error) from the OPLS-DA supervised model discriminating protein hydrolysate C vs. D.

Supplementary Table 7 Differential compounds as resulted from Volcano Plot analysis (ANOVA *P* < 0.01, fold-change > 10) **(A)** for protein hydrolysate C and D separately, at 0.001%, compared to control, **(B)** for protein hydrolysate C and D separately, at 0.1% and **(C)** under 150 mM NaCl stress, compared to untreated control. Compounds are reported with fold-change values, ontology, SMILES and InChIKey annotations.
