# Supplementary figures and images for: Seed Priming With Protein Hydrolysates Improves Arabidopsis Growth and Stress Tolerance to Abiotic Stresses
Source: Front Plant Sci. 2021 Jun 8;12:626301. doi: 10.3389/fpls.2021.626301 (PMC8218911; doi:10.3389/fpls.2021.626301)

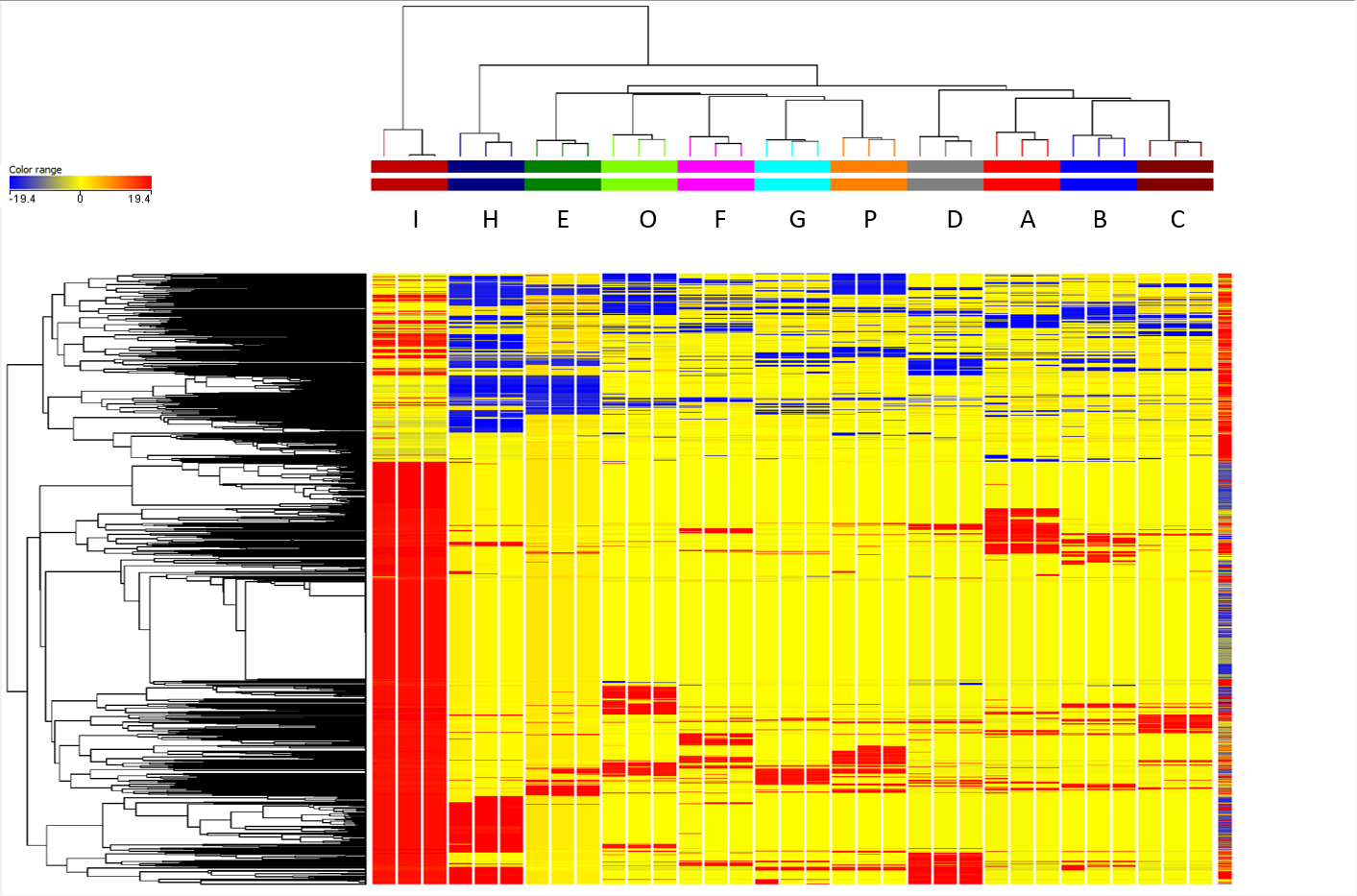

Supplement: Supplementary Figure 1 — Hierarchical cluster analysis conducted on the phytochemical composition of the different PHs; a fold-change heat map was done, and Euclidean distance used for clustering. [file Image_1.TIF]

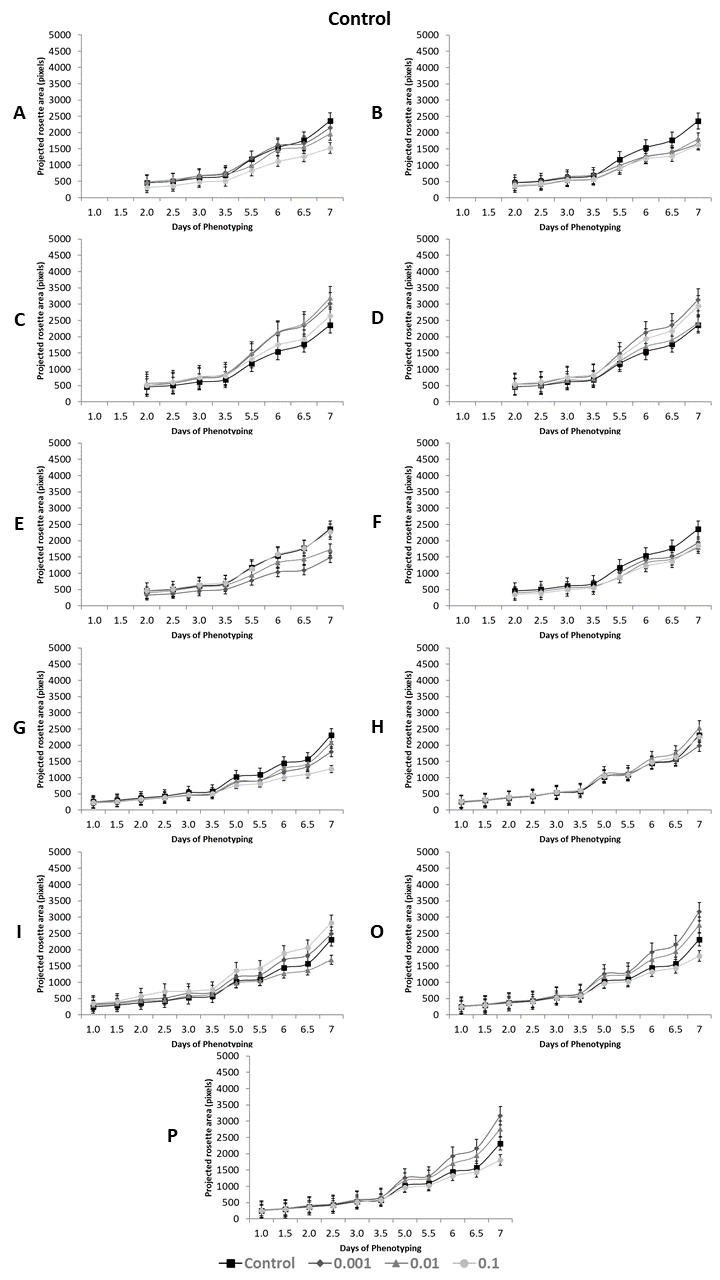

Supplement: Supplementary Figure 2 — Growth of the plants in control conditions following the priming with the set of protein hydrolysates. Projected rosette area (pixels) of Arabidopsis seedlings primed with the 11 protein hydrolysates (A–P) at three concentrations (0.001, 0.01, and 0.1 μl/ml) and grown for 7 days in 48-well plates under control conditions. Rosette area was extracted from RGB images acquired twice a day (am and pm) over the period of 1 week. Values represent the average of the 96 biological replicates per treatment, bars represent SE. [file Image_2.PNG]

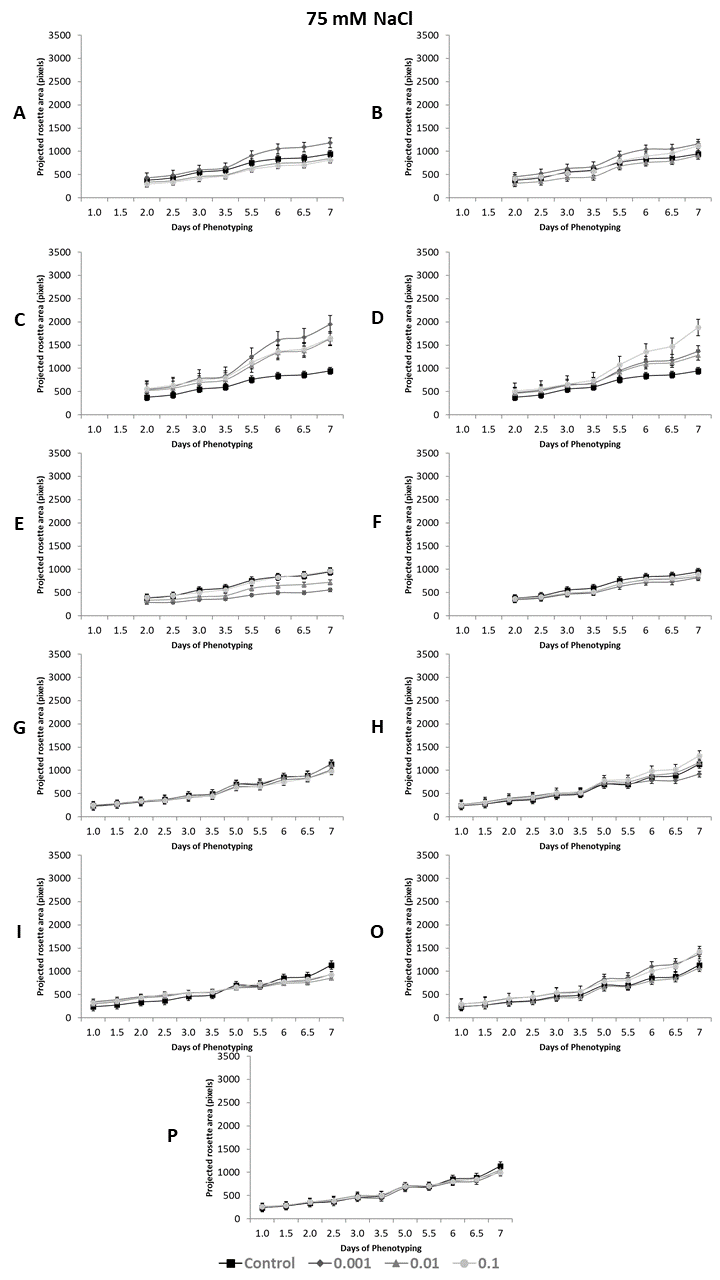

Supplement: Supplementary Figure 3 — Growth of the plants in moderate salt stress conditions following the priming with the set of protein hydrolysates. Projected rosette area (pixels) of Arabidopsis seedlings primed with the 11 protein hydrolysates (A–P) at three concentrations (0.001, 0.01, and 0.1 ml/ml) and grown for 7 days in 48-well plates under moderate (75 mM NaCl) salt stress conditions. Rosette area was extracted from RGB images acquired twice a day (am and pm) over the period of 1 week. Values represent the average of the 96 biological replicates per treatment, bars represent SE. [file Image_3.PNG]

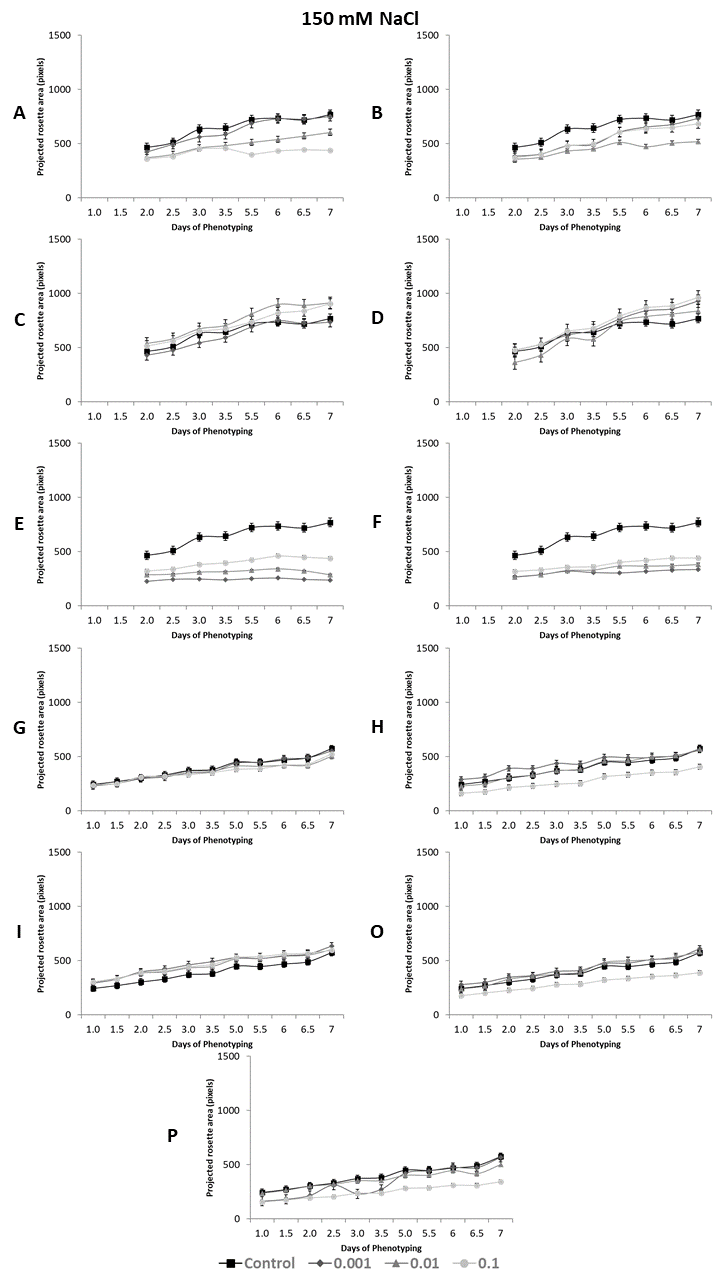

Supplement: Supplementary Figure 4 — Growth of the plants in severe salt stress conditions following the priming with the set of protein hydrolysates. Projected rosette area (pixels) of Arabidopsis seedlings primed with the 11 protein hydrolysates (A–P) at three concentrations (0.001, 0.01, and 0.1 ml/ml) and grown for 7 days in 48-well plates under severe (150 mM NaCl) salt stress conditions. Rosette area was extracted from RGB images acquired twice a day (am and pm) over the period of 1 week. Values represent the average of the 96 biological replicates per treatment, bars represent SE. [file Image_4.PNG]

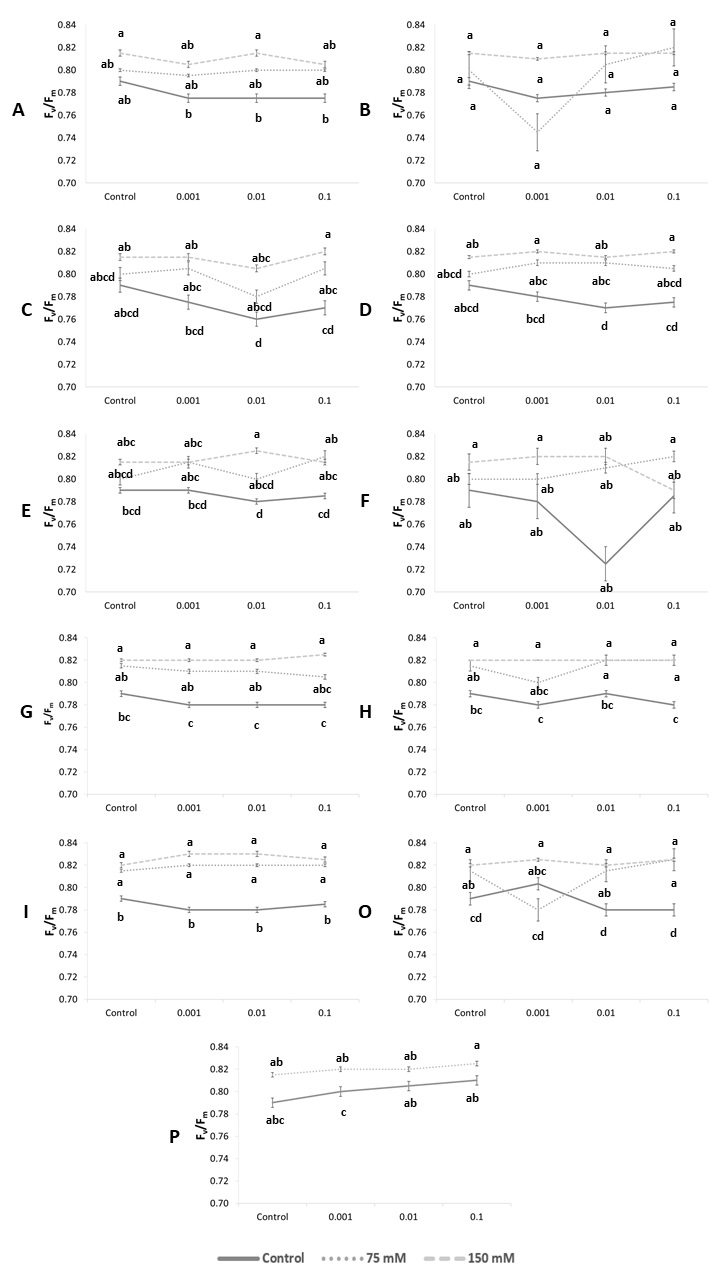

Supplement: Supplementary Figure 5 — Maximum quantum yield of PSII photochemistry in the dark-adapted state (Fv/Fm) of the Arabidopsis seedlings. Graphs show the maximum quantum yield of the plantlets after 7 days of in control, moderate (75 mM NaCl), and severe (150 mM NaCl) salt stress conditions. Seedlings were primed with the 11 protein hydrolysates at three concentrations (0.001, 0.01, and 0.1 ml/ml). Values represent the average of the 96 biological replicates per treatment, bars represent SE. Different letters are used to indicate the significant differences between the treatments (different PH’s and control treatment) using post hoc Tukey’s test (p < 0.05). [file Image_5.jpg]
